# Clinical Trial Participation Motivation: Role of Smoking Status

**DOI:** 10.3390/healthcare13040389

**Published:** 2025-02-11

**Authors:** Chidubem Egboluche, Rifath Ara Alam Barsha, Shervin Assari, Payam Sheikhattari

**Affiliations:** 1 School of Community Health & Policy, Morgan State University, Baltimore, MD 21251, USA; chegb4@morgan.edu (C.E.); payam.sheikhattari@morgan.edu (P.S.); 2 School of Nursing, John Hopkins University, Baltimore, MD 21205, USA; rbarsha1@jh.edu; 3 Department of Internal Medicine, Charles R. Drew University of Medicine and Science, Los Angeles, CA 90059, USA

**Keywords:** trials, smoking, motivation, ethnic groups, population groups

## Abstract

**Introduction**: The success of clinical trials hinges on the effective recruitment and retention of participants, which remains a persistent challenge. Smoking has well-documented adverse effects on health and is a significant predictor of various chronic diseases. However, smoking status impact on the motivation to participate in clinical trials is less clear. **Methods**: This cross-sectional study, utilizing data from the Health Information National Trends Survey (HINTS) 5 Cycle 4 with a sample of 3793 participants, investigates how smoking status (current, former, and never smoker) influences motivation to participate in clinical trials using a structural equation model. Key predictors of trial participation include age, gender, education level, race/ethnicity, income, ethnicity, depression, and chronic respiratory conditions. **Results**: In the overall sample, 51.2% of the participants were females, 76.0% were White adults, 83.1% were non-Hispanic, 39.0% had some college education, and 42.5% had a household income of $75,000 or more. The mean age of the participants was 48.4 years, and the mean depression score was 2.2. Structural equation model results showed a significant positive association between female gender and motivation in clinical trial participation for current smokers. For former smokers, older age and Hispanic ethnicity showed negative associations, while education showed a positive association. For those who have never smoked, older age and other races showed negative associations. **Conclusions**: This study highlights the significant role of education, age, gender, and race/ethnicity among people with different smoking statuses in motivating clinical trial participation. Tailored strategies that address these barriers are essential for improving recruitment and retention in tobacco cessation trials.

## 1. Introduction

Clinical trial success relies upon enrolling enough participants and keeping them engaged until the study concludes. Surprisingly, recruiting and retaining participants in clinical trials is an ongoing challenge that significantly impacts the validity, generalizability, and cost of research endeavors [1,2]. Insufficient enrollment and high attrition rates are pervasive issues encountered by investigators across diverse fields of study, often resulting in delays [3]. Global data analysis of all terminated trials within the Clinical Trial Database reported that 55% of trials were terminated due to low accrual rate, making this the single most common reason [4]. Nearly 80% of clinical trials fail to meet their enrolment timelines, and up to 50% of research sites enroll one or no patients [5]. The recruitment difficulties lead to delays of 1 to 6 months in most clinical trials. These challenges not only prolong study timelines but also result in substantial financial losses for the pharmaceutical industry, estimated at $600,000 to 8 million per day of delay [5]. Tobacco cessation trials, in particular, grapple with recruitment and retention challenges due to the addictive nature of smoking and the complexities of quitting [6].

Numerous studies have identified key predictors of clinical trial participation. These predictors can be broadly categorized into demographic factors, such as age, gender, race/ethnicity, and socio-economic status (SES), and psychological factors, including perceived severity of the condition being studied and perceived effectiveness of the intervention [7,8]. Additionally, logistical factors such as the convenience of trial locations and the time commitment required also play significant roles [9]. Several studies reported that among the demographic factors, age and sex significantly influence trial participation [10,11]. Patients aged 65 or older were significantly less likely to participate compared to younger patients, both in univariate and multivariate analyses. Similarly, men were less likely to participate than women overall [10]. SES influences and reflects a wide range of life outcomes, including access to resources, health, educational attainment, and overall quality of life. Although lower SES is associated with decreased rates of clinical trial enrollment, individuals with medical conditions are more likely to participate in clinical trials due to the potential for direct medical benefits [12,13]. Smoking has well-documented adverse effects on health and is a significant predictor of various chronic diseases [14]. However, studies that focus on clinical trial participation have tended to emphasize disease-specific factors rather than behavioral factors like smoking, thereby neglecting the potential for smoking status to act as a confounding variable in trial outcomes and participation rates [9,13]. This omission can lead to an incomplete picture of participant characteristics and may skew the generalizability of trial findings if smokers, who represent a significant portion of the population, are underrepresented or mischaracterized. Additionally, studies have typically grouped smokers with other high-risk populations without differentiating between the nuances of their health behaviors and perceptions [15]. This broad categorization misses the opportunity to explore how the stigma associated with smoking or concerns about health risks may uniquely impact smokers’ willingness to participate in clinical trials. Current smokers may have different motivations compared to former smokers or those who have never smoked. Therefore, the impact of smoking status on the motivation to participate in clinical trials is less clear.

Examining these variations can provide insights into how smoking status influences clinical trial participation and help develop more targeted and effective recruitment strategies. Understanding these dynamics is crucial, particularly as smoking remains a prevalent issue globally, affecting millions of people and posing significant public health challenges [16,17].

Tobacco cessation research is particularly affected by difficulties in recruiting and retaining study participants. Smoking cessation is a complex process that often requires multiple attempts and long-term follow-up to achieve success [18]. Participants fail to complete tobacco cessation programs, with completion rates below 50% [19]. The low recruitment and retention rates can compromise the interpretation of study results and limit the generalizability of findings. Furthermore, the underrepresentation of diverse demographics, such as with respect to sex, socioeconomic status, and race/ethnicity, in clinical trials perpetuates health disparities and limits the applicability of research findings [20]. Despite the significant burden of smoking-related morbidity and mortality, previous studies reveal a striking disparity in clinical trial representation, with White participants constituting approximately 90% of participants in smoking cessation trials and other studies conducted in the United States and Canada [18,21]. This trend persists across trials conducted exclusively in the United States, where Black and other ethnic minority groups remain significantly underrepresented. In a 2020 study by Pepperrell et al., women, especially women of color, were significantly underrepresented in HIV treatment, vaccine, and cure studies, with Black women being 35% underrepresented relative to HIV prevalence in phase 3 trials of modern antiretrovirals. This underrepresentation hinders the comprehensive evaluation of sex-related differences in treatment efficacy and potential adverse effects. Hence, there is a pressing need for tailored recruitment and retention strategies, especially for underserved populations, to ensure the inclusivity and effectiveness of clinical research. This lack of diversity is especially concerning in tobacco research, as smoking prevalence and cessation outcomes often vary across these demographic groups [22]. Addressing these disparities in enrollment and retention is crucial for ensuring the generalizability and equitable impact of tobacco cessation interventions.

More recently, the Health Information National Trends Survey (HINTS) has emerged as a promising avenue to address the complexities of enrollment and retention in clinical trials while examining motivational factors across diverse population characteristics. With its comprehensive data on demographics, social determinants, health status, health-information-seeking behaviors, and health beliefs, HINTS provides a unique opportunity to understand the motivations underlying research participation and to develop targeted recruitment and retention strategies. By leveraging HINTS data, this study can gain insights into the factors influencing willingness to participate in clinical trials by current smokers, never smokers, and former smokers. This will inform best practices for enrollment and retention among various demographic groups, thereby enhancing the effectiveness and inclusivity of tobacco cessation interventions. Thus, we use HINTS data to investigate our hypothesis, and the findings have significant potential. Our specific aim is to identify the key motivational factors for participation in clinical trials among current smokers, former smokers, and never smokers using data from the HINTS database. Toward this aim, we hypothesize that motivational factors for participating in clinical trials will differ significantly among current smokers, former smokers, and never smokers.

## 2. Methods

### 2.1. Data Source

This study utilized data from the Health Information National Trends Survey (HINTS) 5 Cycle 4 (*n* = 3793) [23,24], a cross-sectional recurring nationally representative survey of the U.S. non-institutionalized adult population (aged 18 years and older) which collects comprehensive data on health-related information and health-related knowledge, attitudes, and behavior, conducted by the National Cancer Institute (NCI). HINTS utilizes a multistage, stratified sampling design using both telephone- and web-based approaches to reach a diverse population in the United States. Cycle 4 of HINTS 5 was conducted only via mail. The data collection for Cycle 4 began on 24 February 2020 and concluded on 15 June 2020. To encourage participation, a $2 pre-paid incentive was offered, and the response rate was 36.6%. Participants were randomly selected to ensure diverse demographic representation. Detailed survey and data collection processes are documented elsewhere. HINTS is a de-identified public-use dataset, exempting this study from full institutional review board review.

### 2.2. Study Sample

The current study consists of adults aged 18 and older of all race/ethnic groups (*n* = 3793).

### 2.3. Outcome Variable

The primary outcome variable was the motivation for participating in clinical trials. This was assessed using a single item: “Imagine that you had a health issue, and you were invited to participate in a clinical trial for that issue. How much would each of the following influence your decision to participate in the clinical trial?” The statements were: (1) “I would be helping other people by participating”, (2) “I would get paid to participate”, (3) “I would get support to participate such as transportation, childcare, or paid time off from work”, (4) “If my doctor encouraged me to participate”, (5) “If my family and friends encouraged me to participate”, (6) “I would want to get better”, (7) “I would get the chance to try a new kind of care”, and (8) “If the standard care was not covered by my insurance”. The response options were “Not at all”/“A little”/“Somewhat”/“A lot”. We coded the response items as 1 = “Not at all”, 2 = “A little”, 3 = “Somewhat”, and 4 = “A lot”, where a higher score indicated higher motivation. These eight items were used in the SEM to create a latent variable (continuous measure), which served as the study’s outcome.

### 2.4. Predictors

The predictors included age, gender (male/female), race (White/Black/others), ethnicity (Hispanic/non-Hispanic), education level (less than high school/high school graduate/some college/college graduate or more), household income (less than $20,000/$20,000 to <$35,000/$35,000 to <$50,000/$50,000 to <$75,000/$75,000 or more), depression, and number of chronic diseases. Other races include Asian, Pacific Islander, American Indian or Alaska Native, and multiple races. The Patient Health Questionnaire-4 (PHQ-4), a self-administered tool, was used to measure depressive symptoms [25]. Each question is rated based on the frequency of symptoms over the past two weeks, with responses including not at all (0), several days (1), more than half the days (2), and nearly every day (3). We calculated the sum of the scores ranging from 0 to 12, where a higher score indicated a more depressive symptom. The variable was used as a continuous measure. To measure number of chronic diseases, HINTS asked participants about the presence or absence of some chronic conditions such as diabetes, high blood pressure, heart disease, lung diseases, and cancer. The sum of these variables was calculated for all the study participants. This variable ranged from 0 to 5 and was used as a continuous measure in the models. Smoking status was evaluated using two questions asked to HINTS participants. The first question was, “Have you smoked at least 100 cigarettes in your entire life?”, and the options were “yes” and “no”. Those who responded “yes” to this question were asked the following question: “How often do you now smoke cigarettes?”, and the response options were “Everyday”/“Some days”/“Not at all”. Those who responded “no” to the first question were labeled as never smokers. Those who responded “Everyday” or “Some days” to the second question were considered current smokers, and those who responded “Not at all” were considered former smokers. The coding was done as follows: 1 = current smoker, 2 = former smoker, 3 = never smoker. We used this variable in the stratified model.

### 2.5. Statistical Analyses

Statistical analysis was performed, adjusting for the complex survey design. The Taylor series linearization variance estimation method provided by HINTS was employed to accommodate complex sample designs and produce nationally representative estimates of statistical measures. We estimated the correlations between the predictor variables to identify and address multicollinearity. The results did not indicate strong multicollinearity; therefore, the correlation matrix is not included. The univariate and bivariate results were presented as weighted proportions and (mean/median with) standard error (SE), interquartile range (IQR), and 95% confidence interval (CI). Stata weighted descriptives automatically generate mean/proportions, SE, and 95% CI, which are corrected for the survey design variables. A practical challenge is that a person’s level of willingness to participate in a clinical trial is not directly observable. Thus, motivation was treated as a latent variable and inferred from observable response items. A structural equation model (SEM) was used to perform the analysis in the pooled sample to identify predictors of motivation in clinical trial participation. In addition, a multi-group SEM with gender, race, and ethnicity was employed to determine the group differences. Smoking status (current, former, and never) was used to estimate the group difference. In the multigroup SEM models, no paths were constrained to be invariant across groups based on smoking status. All paths were allowed to vary across the smoking status groups, enabling the estimation of group-specific relationships. The results of factor analysis are shown as Tables S1 and S2 in the Supplementary Materials. The results of the SEM were presented as beta coefficient (β), SE, and *p* values. The significance level was set at *p* ≤ 0.05. Stata 15.0 (Stata Corp, College Station, TX, USA: StataCorp LLC) was used for the analysis.

## 3. Results

Table 1 presents the descriptive statistics of the overall sample and by smoking status. In the overall sample, 51.2% the participants were females, 76.0% were White adults, 83.1% were non-Hispanic, 39.0% had some college education, and 42.5% had a household income of $75,000 or more. The median age of the participants was 49 years, and the median depression score was 1.0. While most current and former smokers were male (55.7% and 57.6%, respectively), most never-smoking adults were female (56.0%). The percentage of college graduates was higher for never smokers (36.9%) compared to current (11.9%) and former (24.4%) smokers. About 25.1% of current smokers reported having a household income of $75,000 or more, compared to 44.4% and 45.7% of former and never smokers, respectively. White and non-Hispanic adults constituted the majority group for all three categories of smoking status. The mean age was different across the three groups. While the median age of former smokers was 57 years, it was 47 years for current smokers and 46 years for never smokers. The median depressive score was 2.0 for current smokers, 1.0 for former smokers, and 1.0 for never smokers. The mean score of the number of chronic diseases was highest for former smokers, at 1.2. The 95% CIs for our variables are reported in Supplementary Materials Figure S2.

Table 2 presents the mean scores, SE, and 95% CI for eight items measuring motivation to participate in clinical trials, alongside standardized beta coefficients (β), their SE, and 95% CIs from the SEM. The item “I would want to get better” had the highest mean score (3.51, SE = 0.02). All items showed significant positive associations with motivation (*p* < 0.001).

Table 3 presents the Spearman correlation coefficients among the study variables. Most correlations are well below ±0.3, with several near-zero values (e.g., age and race (Black): ρ = 0.00), indicating no strong linear dependencies. Overall, the correlations are low to moderate, with no values exceeding ±0.7, suggesting that severe multicollinearity is unlikely.

Table 4 shows the SEM results of the pooled sample and by smoking status (Supplementary Materials Figure S1). For the pooled sample, older age (β = −0.114, *p* < 0.05) and other races (β = −0.105, *p* < 0.01) showed significant negative associations with motivation in clinical trial participation. A significant positive association was found between education (β = 0.136, *p* < 0.001) and motivation in clinical trial participation. The model fit shows that the coefficient of determination is 0.06.

SEM results by smoking status show a significant positive association between female gender (β = 0.155, *p* < 0.05) and motivation in clinical trial participation for current smokers. Among former smokers, older age (β = −0.137, *p* < 0.05) and Hispanic ethnicity (β = −0.134, *p* < 0.05) show significant negative associations with motivation. Additionally, a significant positive association with education (β = 0.192, *p* < 0.001) is observed. For those who have never smoked, older age (β = −0.116, *p* < 0.05) and other races (β = −0.126, *p* < 0.01) exhibited significant negative associations, whereas education (β = 0.139, *p* < 0.001) showed significant positive association with motivation to participate in clinical trials. The model fit shows that the coefficient of determination is 0.08.

Table 5 presents the SEM results for the pooled sample, with smoking status included as a variable (Supplementary Materials Figure S2). Older age (β = −0.121, *p* < 0.05) and being of other races (β = −0.101, *p* < 0.01) showed significant negative associations with motivation for clinical trial participation. Higher education demonstrated a significant positive association (β = 0.142, *p* < 0.001). The model fit indicates a coefficient of determination of 0.06.

## 4. Discussion

This study aimed to identify the key factors influencing motivation for participation in clinical trials among current smokers, former smokers, and never smokers using data from the HINTS. Our analysis revealed that education has a significant positive association with motivation to participate in clinical trials, while older age and belonging to other racial groups (Asian, Pacific Islander, American Indian or Alaska Native, and multiple races) showed significant negative associations. The motivational factors for participating in a clinical trial varied significantly among different smoking statuses.

The relationship between SES and motivation to participate in clinical trials has been extensively studied, with various aspects of SES, such as education and income, often showing different effects. In this study, we found that only educational attainment, and not income, had a significant association with motivation to participate in clinical trials. This finding suggests that SES exerts a differential effect, where understanding and knowledge, as indicated by education level, play a more crucial role than material wealth in influencing clinical trial participation. This result is consistent with [26], which noted that educated individuals were more likely to participate in clinical research, and [27], which reported that 97% of their participants were college educated, showing favorable views of research and willingness to engage in clinical trials. Income, which reflects material wealth, did not show a significant association with motivation to participate in clinical trials in our study. While income can affect access to healthcare and overall health status, it may not directly influence an individual’s cognitive abilities to understand and engage with clinical research [28]. This finding aligns with previous research suggesting that material resources alone do not necessarily translate into higher motivation or capability to participate in clinical trials [28]. Our study reveals that this association is not significant among current smokers, whereas it is significant among former and never smokers. Hence, current smokers may present unique barriers that education alone does not address. One previous study emphasizes that younger smokers, African Americans, and those with low socioeconomic status are less likely to enroll in cessation trials [29]. Although current smokers demonstrate a high rate of seeking health information, younger smokers may not perceive their smoking as a serious health risk [29,30]. These factors might mitigate the otherwise positive effects of higher education on clinical trial participation seen in other groups. Former smokers, having successfully quit, might be more health-conscious and thus more motivated by educational interventions to participate in clinical trials [28]. Never smokers, on the other hand, might not face the same health challenges as current smokers, allowing education to play a more direct role in their motivation to engage in clinical research [14].

The study also examined the influence of demographic factors such as age, ethnicity, race, and gender on the motivation to participate in clinical trials, revealing nuanced and significant associations across different smoking statuses. Our findings show a significant negative association between age and motivation to participate in clinical trials among former smokers and never smokers but not among current smokers. Our result aligns with [10], which stated that older age was consistent with poor accrual by univariate and multivariate analysis and that these participants were less likely to be current smokers [11]. This suggests that older individuals in the former- and never-smoker categories may be less motivated to engage in clinical trials, potentially due to increased health concerns, perceived risks, or a lack of perceived benefit from participation [31]. However, this association is not observed in current smokers, indicating that factors other than age might play a more pivotal role in influencing their motivation. Smoking often begins in adolescence, leading to long-term dependence and potentially diminishing the perceived benefits of quitting at an older age. This suggests that for long-term smokers, age might be less of a deciding factor in their willingness to participate in cessation programs or trials, as they might believe that they are less susceptible to the risks, downplay the health risks associated with smoking, or believe that any damage is irreversible [32]. Current smokers might face unique barriers to clinical trial participation, like lower socioeconomic status or mistrust in the medical community, that are unrelated to age and might supersede any potential negative influence of age on their motivation. Studies emphasize that lower socioeconomic status, often correlated with lower levels of education and health literacy, can significantly hinder clinical trial enrollment.

Ethnicity also plays a significant role. There is a significant negative association between being Hispanic and motivation to participate in clinical trials among former smokers, while this is not significant among current smokers or never smokers. This could be attributed to cultural factors, language barriers, or historical mistrust in medical research prevalent in Hispanic communities [33]. Generally, this study aligns with the study of Warren et al. (2002) [34], which reported that Asian-American and Hispanic adults were accrued to clinical trials at lower rates than White cancer patients of the same age irrespective of smoking status.

Race, particularly with regard to individuals identifying as races other than Black and White, shows a significant negative association with motivation to participate in clinical trials among never smokers but not among current or former smokers. This indicates that members of racial minorities who have never smoked might face systemic healthcare disparities, perceived discrimination, or lack of targeted outreach efforts, which diminish their motivation to participate [35]. Current smokers do not show this negative association, which might suggest that their immediate health concerns related to smoking could override racial barriers. This aligns with a study that found Black patient participation in clinical trials comparable to White participation in some evaluations [10]. Several studies highlight that individuals with medical conditions, particularly those related to smoking, might be more motivated to participate in clinical trials due to the potential for direct medical benefits, suggesting that the inherent health concerns associated with smoking might be a stronger motivator for current smokers than for never smokers, potentially overshadowing the influence of race [12,13,28]. Former smokers, while not showing a significant association, may have experiences or health motivations post-cessation that impact participation willingness [16].

Gender has been found to be a significant predictor of clinical trial participation [10]. A study by Saphner et al. [10] reveals that women are more likely to participate in clinical trials, which aligns with the results of our study revealing a significant effect in the stratified model. The association between gender and motivation to participate in clinical trials, particularly among current smokers, appears to be complex and may be influenced by various factors such as the specific health condition being studied and the type of trial [36]. Being female is significantly associated with motivation to participate in clinical trials among current smokers but not among former and never smokers. This significant association in current smokers could be due to gender-specific health concerns or motivations, such as the heightened awareness of smoking-related health risks among women during pregnancy, leading to a higher motivation to engage in clinical trials as a proactive health measure [37]. For former smokers, gender does not significantly influence motivation, suggesting that other demographic or psychosocial factors like the fear of relapse, managing lingering health risk, SES, and pregnancy and breastfeeding are major motivators for female former smokers [16].

By examining the nuances of how smoking status influences clinical trial participation, this study’s findings fill a gap in the literature and provide valuable insights for designing targeted recruitment strategies that address the unique barriers and motivations of current, former, and never smokers with respect to smoking cessation trials. This focus is particularly important in ensuring that clinical trials are inclusive and that their findings are generalizable across diverse populations.

The next step should involve using a mixed-methods study to explore and understand how age, education, gender, and race predict motivation to participate in clinical trials. This will help us learn how they predict motivation toward clinical trial participation among the different smoking statuses. Additionally, there should be a qualitative study to identify barriers faced by older adults and individuals of different racial backgrounds. Understanding these barriers through qualitative studies could inform more effective, tailored recruitment strategies.

Policymakers should create supportive environments for clinical trial participation through policy interventions. This includes funding for educational programs through the creation of special grants and services focusing on clinical trial improvement. Financial incentives for participation should include transportation support and childcare services. Policies that specifically address the needs of racial minorities will help ensure more inclusive and representative participation in clinical trials.

Researchers should implement recruitment strategies that include personalized communication aimed at building trust and reducing perceived risks among current smokers. Utilizing peer support systems, whereby former smokers share their positive experiences with clinical trial participation, can also be effective. Incentives, clear communication, and community engagement are crucial to improving participation rates [4].

This study’s strengths include the use of a large, nationally representative dataset and the application of an SEM to understand complex relationships between variables. However, while providing valuable insights into the smoking behaviors of the population, the study is not without its limitations. The distribution of our sample size among current smokers (*n* = 436), former smokers (*n* = 935), and never smokers (*n* = 2422) introduces several constraints that must be considered when interpreting the findings. The smaller number of current smokers compared to former and never smokers may reduce the statistical power of analyses within this group, potentially leading to less robust conclusions about current smoking behaviors and associated health outcomes. Additionally, the large sample of never smokers may skew overall results, possibly underestimating the risks faced by current and former smokers. The use of a cross-sectional design limits the ability to infer causality, because it provides only a snapshot in time in terms of data. Additionally, self-reported data may be subject to bias, such as recall bias, response bias, and social desirability bias. The retrospective categorization of former smokers introduces recall bias, as participants may inaccurately report past smoking habits, affecting data reliability. The response rate for HINTS Cycle 4 was 36.6%, which is relatively low. A low response rate can introduce non-response bias if the individuals who chose not to respond differ significantly from those who did [38]. This can affect the representativeness and reliability of the results. Individuals may provide responses that they believe are more socially acceptable or favorable, rather than their true thoughts or behaviors. This can lead to overreporting of positive behaviors and underreporting of negative ones. The predominance of one racial group (White, 76%; other race, 24%) might skew the findings in favor of the experiences or perspectives of the predominant group, potentially overlooking the experiences of the minority groups. Moreover, there are differences across studies in how motivation for clinical trial participation is measured. For instance, in some countries, participants can legally receive payment for enrolling in clinical trials, while in some other countries, payment for trial enrollment is strictly prohibited, undermining the generalizability of the measurement of motivation for clinical trial participation. Furthermore, there were differences in sampling probabilities and oversampling strategies between men and women, which highlights the importance of interpreting weighted results within the context of survey methodology. In addition, the interpretation of the results should consider the potential influence of random error and random fluctuation, which could have affected the estimates in both the overall and group-specific models. Additionally, collider stratification bias may have arisen due to the conditioning on certain variables.

Given the limitations of the current study, future research should explore several avenues to better understand the roles of educational attainment, age, gender, and race as predictors of motivation to participate in clinical trials. Qualitative studies could offer deeper insights into the specific barriers and facilitators that current smokers face regarding clinical trial participation, and could inform the development of targeted interventions aimed at increasing clinical trial participation among current smokers.

## 5. Conclusions

In conclusion, this study highlights the significant roles of education, age, gender, and race among people with different smoking statuses (current smoker, former smoker, and never smoker) in motivating clinical trial participation. Tailored strategies that address these barriers are essential for improving recruitment and retention in tobacco cessation trials. By leveraging insights from the HINTS data, researchers and policymakers can develop more effective interventions to enhance participation and ensure the success of clinical trials.

## Figures and Tables

**Table 1:** Descriptive statistics of the overall sample (*n* = 3793) and by smoking status.

	Current Smoker (*n* = 436)	Former Smoker (*n* = 935)	Never Smoker (*n* = 2422)	Total = 3793
Variables	*n* (%)	*n* (%)	*n* (%)	*n* (%)
Sex ***				
Male	196 (55.7)	456 (57.6)	890 (44.0)	1542 (48.8)
Female	231 (44.3)	462 (42.4)	1478 (56.0)	2171 (51.2)
Education ***				
Less than High School	51 (12.7)	64 (7.9)	153 (7.1)	268 (8.1)
High School Graduate	112 (27.0)	184 (24.8)	393 (20.5)	689 (22.4)
Some College	163 (48.4)	307 (42.9)	596 (35.5)	1066 (39.0)
College Graduate or More	99 (11.9)	358 (24.4)	1197 (36.9)	1654 (30.5)
Household Income ***				
Less than $20,000	136 (23.8)	134 (12.7)	343 (13.9)	613 (15.1)
$20,000 to <$35,000	60 (13.9)	115 (10.8)	270 (11.2)	445 (11.5)
$35,000 to <$50,000	43 (13.6)	120 (14.0)	289 (11.9)	452 (12.6)
$50,000 to <$75,000	77 (23.6)	148 (18.1)	362 (17.3)	587 (18.3)
$75,000 or More	82 (25.1)	337 (44.4)	893 (45.7)	1312 (42.5)
Race ***				
White	300 (76.6)	718 (85.9)	1561 (72.2)	2579 (76.0)
Black	73 (13.5)	114 (7.8)	399 (15.1)	586 (13.2)
Others	36 (9.9)	57 (6.3)	270 (12.7)	363 (10.8)
Ethnicity ***				
Not Hispanic	346 (87.6)	750 (90.9)	1782 (79.2)	2878 (83.1)
Hispanic	57 (12.4)	98 (9.1)	437 (20.8)	592 (16.9)
	Median	Median	Median	Median
Age *	47	57	46	49
Depression	2.0	1.0	1.0	1.0
	Mean	Mean	Mean	Mean
Number of Chronic Diseases *	0.9	1.2	0.7	0.8

Note: significance *** *p* < 0.001, * *p* < 0.05.

**Table 2:** Loadings of the eight items used to measure motivation.

	**Mean**	**SE**	**95% CI**
I would be helping other people by participating	2.86	0.03	2.80–2.93
I would get paid to participate	2.54	0.03	2.48–2.61
I would get support to participate such as transportation, childcare, or paid time off from work	2.50	0.03	2.44–2.57
If my doctor encouraged me to participate	2.84	0.03	2.79–2.90
If my family and friends encouraged me to participate	2.65	0.03	2.60–2.71
I would want to get better	3.51	0.02	3.47–3.56
I would get the chance to try a new kind of care	2.84	0.03	2.78–2.90
If the standard care was not covered by my insurance	2.93	0.04	2.85–3.01
	**Beta**	**SE**	**95% CI**
I would be helping other people by participating	0.67 ***	0.01	0.65–0.69
I would get paid to participate	0.44 ***	0.01	0.41–0.47
I would get support to participate such as transportation, childcare, or paid time off from work	0.51 ***	0.01	0.48–0.54
If my doctor encouraged me to participate	0.75 ***	0.01	0.73–0.76
If my family and friends encouraged me to participate	0.70 ***	0.01	0.68–0.72
I would want to get better	0.77 ***	0.01	0.75–0.79
I would get the chance to try a new kind of care	0.71 ***	0.01	0.69–0.73
If the standard care was not covered by my insurance	0.59 ***	0.01	0.57–0.61

Note: SE stands for standard error, CI stands for confidence interval, *** *p* < 0.001.

**Table 3:** Spearman correlation coefficients among study variables.

Variables	1	2	3	4	5	6	7	8	9
1. Age	1.00								
2. Female	−0.07 ***	1.00							
3. Education	−0.16 ***	−0.01	1.00						
4. Household Income	−0.19 ***	−0.10 ***	0.44 ***	1.00					
5. Black	0.00	0.05 **	−0.06 **	−0.14 ***	1.00				
6. Other	−0.09 ***	−0.03	0.04 *	−0.01	−0.13 ***	1.00			
7. Hispanic	−0.12 ***	0.01	−0.21 ***	−0.11 ***	−0.12 ***	0.01	1.00		
8. Depression	−0.19 ***	0.12 ***	−0.05 **	−0.13 ***	−0.03	0.02	0.03	1.00	
9. Number of Chronic Diseases	0.48 ***	−0.04 *	−0.16 ***	−0.19 ***	0.09 ***	−0.04 *	−0.08 ***	0.06 **	1.00

Note: * *p* < 0.05, ** *p* < 0.01, *** *p* < 0.001.

**Table 4:** Summary of the SEM results in pooled sample and by smoking status (*n* = 3793).

Variables	Overall Motivation in Clinical Trial Participation							
	Pooled Sample		Current Smoker		Former Smoker		Never Smoker	
	Beta	SE	Beta	SE	Beta	SE	Beta	SE
Age (years)	−0.114 *	0.046	−0.105	0.076	−0.137 *	0.056	−0.116 *	0.051
Gender (Female)	0.031	0.034	0.155 *	0.073	0.093	0.051	−0.018	0.042
Education (1–4)	0.136 ***	0.031	0.025	0.068	0.192 ***	0.048	0.139 ***	0.035
Household Income (1–5)	0.035	0.036	0.085	0.085	0.033	0.054	0.026	0.051
Race (Black)	−0.039	0.029	−0.068	0.082	−0.009	0.041	−0.034	0.034
Race (Other)	−0.105 **	0.032	0.003	0.080	−0.096	0.077	−0.126 **	0.039
Ethnicity (Hispanic)	−0.063	0.036	0.094	0.057	−0.134 *	0.051	−0.065	0.045
Depression	0.057	0.034	0.005	0.097	0.035	0.060	0.060	0.040
Number of Chronic Diseases	0.057	0.030	0.129	0.082	0.008	0.067	0.059	0.032

Note: * *p* < 0.05, ** *p* < 0.01, *** *p* < 0.001.

**Table 5:** Summary of the SEM results with smoking status as variables in the pooled sample.

Variables	Motivation for Clinical Trial Participation	
	Beta	SE
Age (years)	−0.121 *	0.046
Gender (Female)	0.037	0.034
Education (1–4)	0.142 ***	0.030
Household Income (1–5)	0.034	0.037
Race (Black)	−0.033	0.029
Race (Other)	−0.101 **	0.032
Ethnicity (Hispanic)	−0.056	0.037
Depression	0.054	0.033
Number of Chronic Diseases	0.052	0.029
Current Smoker	0.018	0.035
Former Smoker	0.052	0.033

Note: * *p* < 0.05, ** *p* < 0.01, *** *p* < 0.001.

## Data Availability

Data are available at https://hints.cancer.gov/data/download-data.aspx (accessed on 1 September 2024).

## Supplementary Materials

The following supporting information can be downloaded at: https://www.mdpi.com/article/10.3390/healthcare13040389/s1, Table S1. Results of Factor Analysis of Motivation Measures Items. Table S2. Detailed Descriptive Statistics of the Overall Sample (*n* = 3793) and by Smoking Status. Figure S1. Summary of structural equation models (SEMs). (a) SEM results in the pooled sample. (b) SEM stratified model, current smoker. (c) SEM stratified model, former smoker. (d) SEM stratified model, never smoker. Note: 1 = I would be helping other people by participating, 2 = I would get paid to participate, 3 = I would get support to participate such as transportation, childcare, or paid time off from work, 4 = If my doctor encouraged me to participate, 5 = If my family and friends encouraged me to participate, 6 = I would want to get better, 7 = I would get the chance to try a new kind of care, 8 = If the standard care was not covered by my insurance. Figure S2. Summary of the SEM results with smoking status as variables in the pooled sample.
