# Supplementary material for: Clinical Trial Participation Motivation: Role of Smoking Status
Source: Healthcare (Basel). 2025 Feb 11;13(4):389. doi: 10.3390/healthcare13040389 (PMC11854915; doi:10.3390/healthcare13040389)
Supplement: Supplementary file 1 [file healthcare-13-00389-s001.zip › healthcare-3320643 -Figures.pdf]

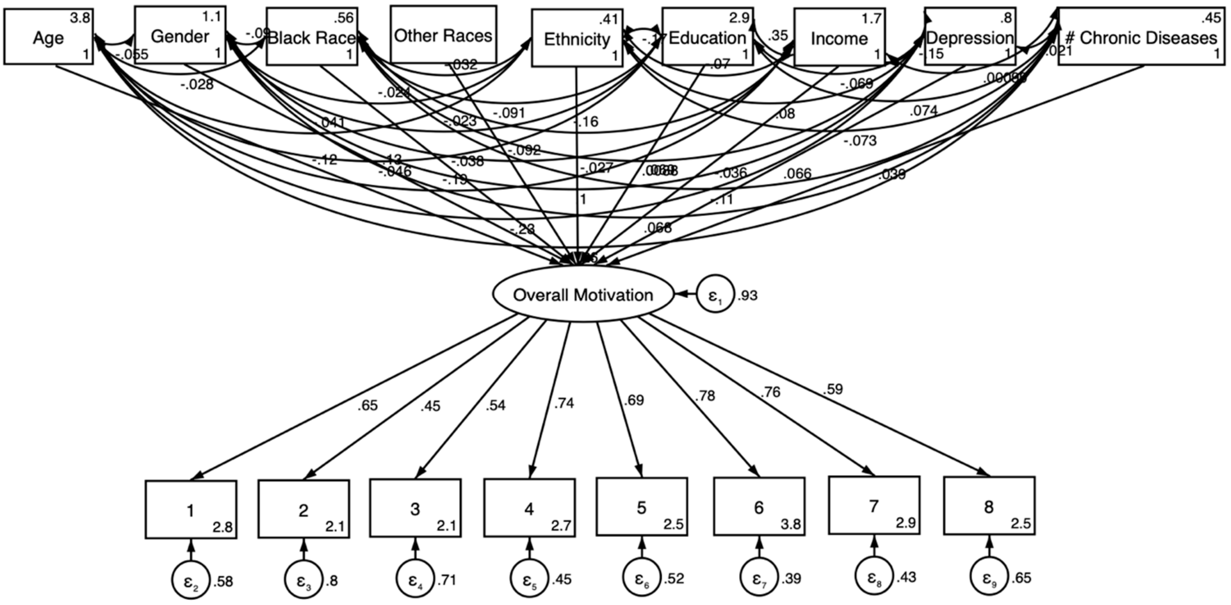
Age
Gender
Black Race
Other Races
Ethnicity
Education
Income
Depression
Chronic Diseases
Overall Motivation
ε1 .93
.65
.45
.54
.74
.69
.78
.76
.59
1 2.8
2 2.1
3 2.1
4 2.7
5 2.5
6 3.8
7 2.9
8 2.5
ε2 .58
ε3 .8
ε4 .71
ε5 .45
ε6 .52
ε7 .39
ε8 .43
ε9 .65

(**a**)

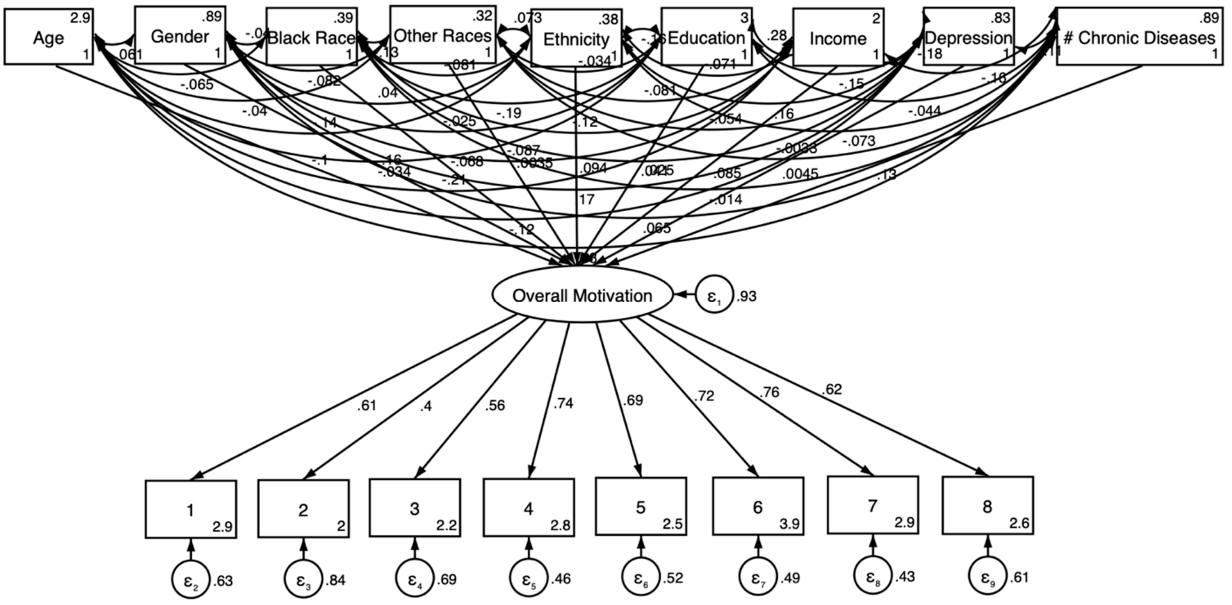
Age
Gender
Black Race
Other Races
Ethnicity
Education
Income
Depression
Chronic Diseases
Overall Motivation
ε1 .93
.61
.4
.56
.74
.69
.72
.76
.62
1 2.9
2 2
3 2.2
4 2.8
5 2.5
6 3.9
7 2.9
8 2.6
ε2 .63
ε3 .84
ε4 .69
ε5 .46
ε6 .52
ε7 .49
ε8 .43
ε9 .61

(**b**)

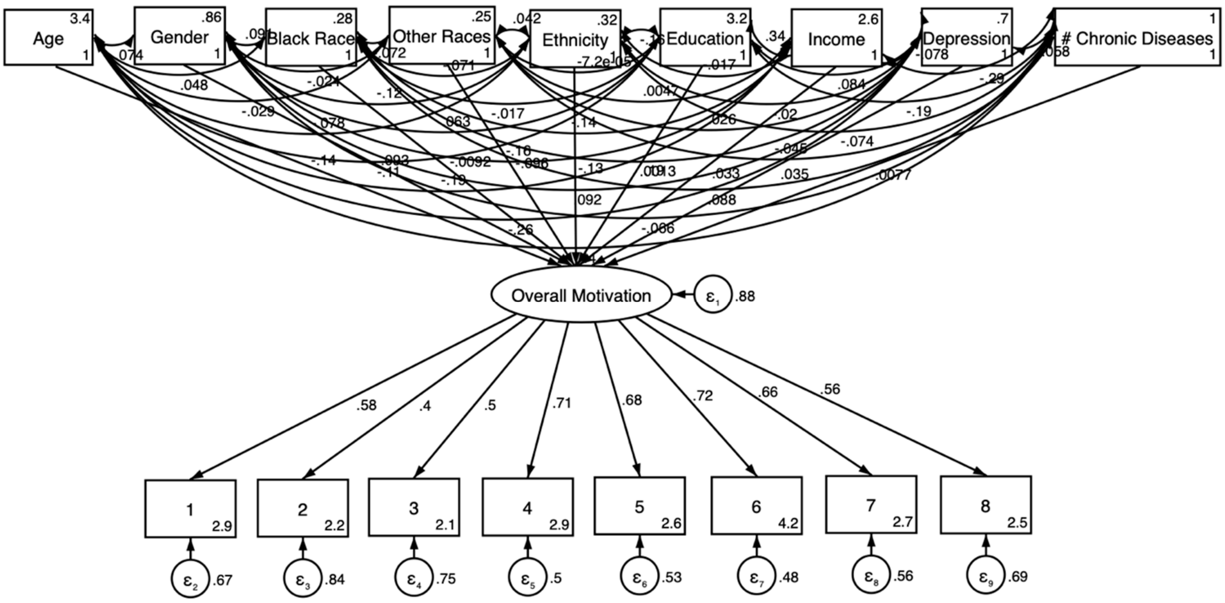

(**c**)

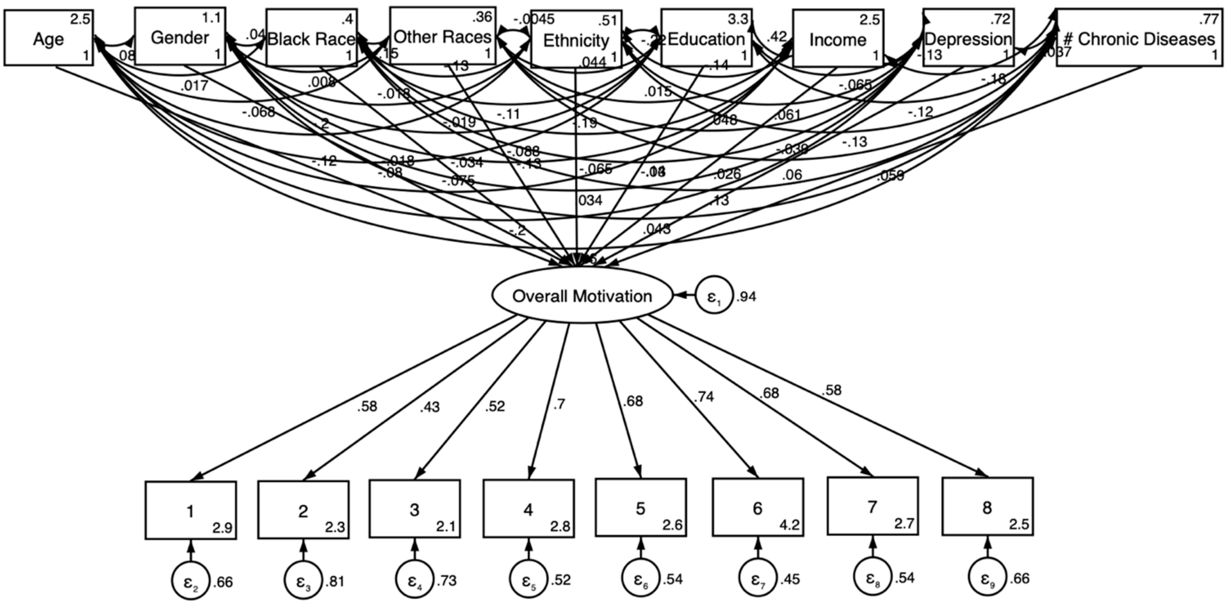

(**d**)

Figure S1. Summary of structural equation models (SEMs). (**a**) SEM results in the pooled sample. (**b**) SEM stratified model, current smoker. (**c**) SEM stratified model, former smoker. (**d**) SEM stratified model, never smoker. Note: 1 = I would be helping other people by participating, 2 = I would get paid to participate, 3 = I would get support to participate such as transportation, childcare, or paid time off from work, 4 = If my doctor encouraged me to participate, 5 = If my family and friends encouraged me to participate, 6 = I would want to get better, 7 = I would get the chance to try a new kind of care, 8 = If the standard care was not covered by my insurance.

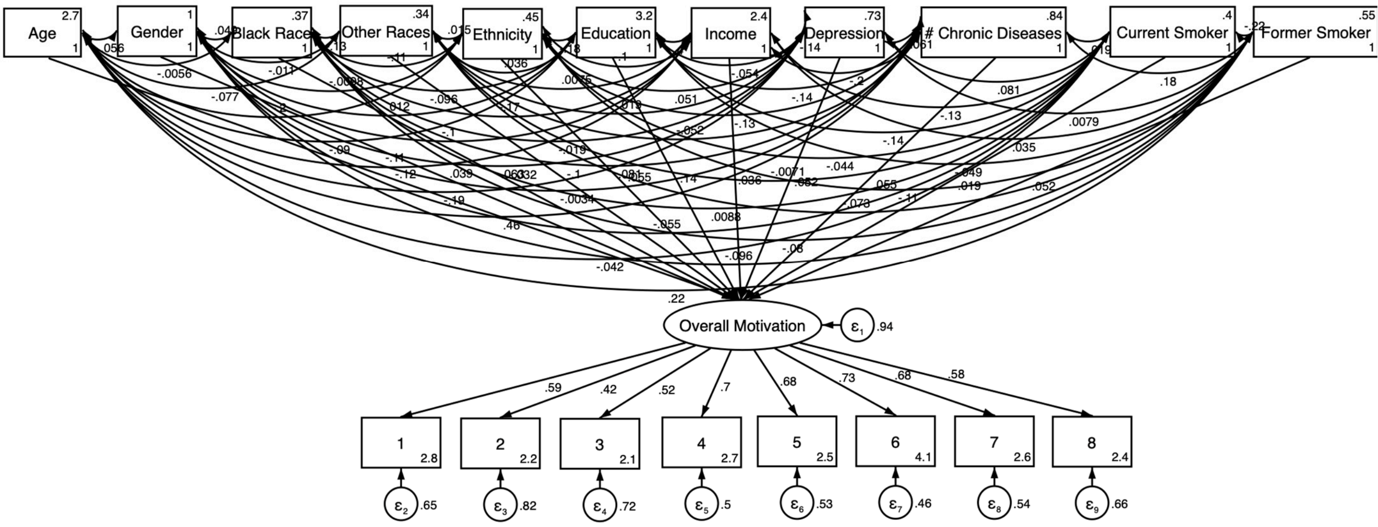

Figure S2. Summary of the SEM results with smoking status as variables in the pooled sample.
