# Supplementary material for: Clinical Trial Participation Motivation: Role of Smoking Status
Source: Healthcare (Basel). 2025 Feb 11;13(4):389. doi: 10.3390/healthcare13040389 (PMC11854915; doi:10.3390/healthcare13040389)
Supplement: Supplementary file 1 [file healthcare-13-00389-s001.zip › healthcare-3320643-Tables.pdf]

Supplemental Materials:

Table S1. Results of Factor Analysis of Motivation Measures Items.

Items	Overall	Current Smoker	Former Smoker	Never Smoker
I would be helping other people by participating	0.6539	0.5996	0.6874	0.6460
I would get paid to Participate	0.4849	0.4881	0.4620	0.4952
I would get support to participate such as transportation, childcare, or paid time off from work	0.5506	0.6200	0.5645	0.5322
If my doctor encouraged me to participate	0.7298	0.7079	0.7252	0.7347
If my family and friends encouraged me to participate	0.6841	0.6570	0.6831	0.6900
I would want to get better	0.7490	0.7543	0.7338	0.7539
I would get the chance to try a new kind of care	0.6943	0.7577	0.6880	0.6833
If the standard care was not covered by my insurance	0.5778	0.6083	0.5729	0.5720

Table S2. Detailed Descriptive Statistics of the Overall Sample (n = 3,793) and by Smoking Status.

Variables	Current Smoker (n= 436)		Former Smoker (n=935)		Never-Smoker (n=2422)		Total= 3793	
	**n (%)**	**95% CI**	**n (%)**	**95% CI**	**n (%)**	**95% CI**	**n (%)**	**95% CI**
Sex***								
Male	196 (55.7)	0.48-0.63	456 (57.6)	0.51-0.64	890 (44.0)	0.40-0.48	1542 (48.8)	0.46-0.52
Female	231 (44.3)	0.37-0.52	462 (42.4)	0.36-0.49	1478 (56.0)	0.52-0.60	2171 (51.2)	0.48-0.54
Education***								
Less than High School	51 (12.7)	0.08-0.19	64 (7.9)	0.05-0.12	153 (7.1)	0.05-0.10	268 (8.1)	0.06-0.10
High School Graduate	112 (27.0)	0.20-0.35	184 (24.8)	0.20-0.30	393 (20.5)	0.18-0.23	689 (22.4)	0.20-0.25
Some College	163 (48.4)	0.40-0.56	307 (42.9)	0.37-0.49	596 (35.5)	0.32-0.39	1066 (39.0)	0.36-0.42
College Graduate or More	99 (11.9)	0.08-0.18	358 (24.4)	0.20-0.29	1197 (36.9)	0.34-0.40	1654 (30.5)	0.28-0.33
Household Income***								
Less than $20,000	136 (23.8)	0.18-0.30	134 (12.7)	0.10-0.16	343 (13.9)	0.11-0.17	613 (15.1)	0.13-0.17
$20,000 to < $35,000	60 (13.9)	0.10-0.20	115 (10.8)	0.08-0.14	270 (11.2)	0.09-0.13	445 (11.5)	0.10-0.13
$35,000 to < $50,000	43 (13.6)	0.08-0.22	120 (14.0)	0.11-0.18	289 (11.9)	0.10-0.14	452 (12.6)	0.11-0.15
$50,000 to < $75,000	77 (23.6)	0.17-0.32	148 (18.1)	0.14-0.23	362 (17.3)	0.14-0.21	587 (18.3)	0.16-0.21
$75,000 or More	82 (25.1)	0.18-0.34	337 (44.4)	0.39-0.50	893 (45.7)	0.42-0.49	1312 (42.5)	0.39-0.46
Race***								
White	300 (76.6)	0.70-0.82	718 (85.9)	0.82-0.89	1561 (72.2)	0.69-0.75	2579 (76.0)	0.74-0.78
Black	73 (13.5)	0.09-0.19	114 (7.8)	0.06-0.10	399 (15.1)	0.13-0.17	586 (13.2)	0.12-0.15
Others	36 (9.9)	0.06-0.16	57 (6.3)	0.04 .09	270 (12.7)	0.10-0.16	363 (10.8)	0.09-0.13
Ethnicity***								
Not Hispanic	346 (87.6)	0.80-0.92	750 (90.9)	0.87-0.94	1782 (79.2)	0.76-0.82	2878 (83.1)	0.81-0.85
Hispanic	57 (12.4)	0.08-0.20	98 (9.1)	0.06-0.13	437 (20.8)	0.18-0.24	592 (16.9)	0.15-0.19
	Median	95% CI	Median	95% CI	Median	95% CI	Median	95% CI
Age*	47	43.08-50.92	57	54.06-59.94	46	44.04-47.96	49	48.02-49.98
Depression	2.0	1.51-2.49	1.0	0.51-1.49	1.0	0.51-1.49	1.0	-
	Mean	95% CI	Mean	95% CI	Mean	95% CI	Mean	95% CI

Number of Chronic Diseases*	0.9	0.76-1.01	1.2	1.06-1.28	0.7	0.65-0.76	0.8	0.79-0.88

Note. SE stands for standard error. IQR stands for interquartile range. Significance *** $p < 0.001$, $p < 0.05$.
